# Synthesis and Biological Activity of Substituted Urea and Thiourea Derivatives Containing 1,2,4-Triazole Moieties

**DOI:** 10.3390/molecules18033562

**Published:** 2013-03-19

**Authors:** Bedia Kocyigit-Kaymakcioglu, Ahmet Ozgur Celen, Nurhayat Tabanca, Abbas Ali, Shabana I. Khan, Ikhlas A. Khan, David E. Wedge

**Affiliations:** 1 Department of Pharmaceutical Chemistry, Faculty of Pharmacy, Marmara University, Haydarpasa 34668, Istanbul, Turkey; E-Mail: ahmozgcelen@hotmail.com; 2 National Center for Natural Products Research, Research Institute of Pharmaceutical Sciences, The University of Mississippi, University, MS 38677, USA; E-Mails: ntabanca@olemiss.edu (N.T.); aali@olemiss.edu (A.A.); ikhan@olemiss.edu (S.I.K.); skhan@olemiss.edu (I.A.K.); 3 Department of Pharmacognosy, School of Pharmacy, The University of Mississippi, University, MS 38677, USA; 4 Natural Products Utilization Research Unit, Agricultural Research Service, United States Department of Agriculture, University, MS 38667, USA; E-Mail: dwedge@olemiss.edu

**Keywords:** thiourea, urea, 1,2,4-triazole, synthesis, fungicide, plant pathogens, larvicides, mosquitoes, anti-inflammatory, cytotoxicity

## Abstract

A series of novel thiourea and urea derivatives containing 1,2,4-triazole moieties were synthesized and evaluated for their antifungal and larvicidal activity. Triazole derivatives **3a**–**e** and **4a**–**e** were synthesized by reacting thiocarbohydrazide with thiourea and urea compounds **1a**–**e** and **2a**–**e**, respectively, in a 130–140 °C oil bath. The proposed structures of all the synthesized compounds were confirmed using elemental analysis, UV, IR, ^1^H-NMR and mass spectroscopy. All compounds were evaluated for antifungal activity against plant pathogens, larvicidal and biting deterrent activity against the mosquito *Aedes aegypti* L. and *in vitro* cytotoxicity and anti-inflammatory activity against some human cell lines. *Phomopis* species were the most sensitive fungi to these compounds. Compounds **1b**, **1c**, **3a** and **4e** demonstrated selectively good activity against *Phomopis obscurans* and only **1b** and **4e** showed a similar level of activity against *P. viticola.* Compound **3d**, with a LD_50_ value of 67.9 ppm, followed by **1c** (LD_50_ = 118.8 ppm) and **3e** (LD_50_ = 165.6 ppm), showed the highest toxicity against *Aedes aegypti* larvae. Four of these compounds showed biting deterrent activity greater than solvent control, with the highest activity being seen for **1c**, with a proportion not biting (PNB) value of 0.75, followed by **1e**, **2b** and **1a**. No cytotoxicity was observed against the tested human cancer cell lines. No anti-inflammatory activity was observed against NF-κB dependent transcription induced by phorbol myristate acetate (PMA) in human chondrosarcoma cells.

## 1. Introduction

Thioureas are important sulphur and nitrogen-containing compounds that have proved to be useful substances in drug research in recent years [1,2,3,4,5,6]. Some urea derivatives possess valuable antituberculosis, antibacterial and anticonvulsant properties [7,8,9,10]. Most of these compounds include heterocyclic rings such as oxadiazoles, thiadiazoles, triazoles, and pyrazoles. It is well known that the 1,2,4-triazole-derived N-bridged heterocycles find applications in the field of medicine, agriculture and industry [11,12,13,14]. The 1,2,4-triazole nucleus has also been incorporated into a wide variety of therapeutically important molecules to transform them into better drugs. Drugs such as fluconazole, itraconazole, and the new generation of triazoles posaconazole, voriconazole, and ravuconazole are the best examples of potent antifungal molecules possessing triazole nuclei [15,16,17].

Thioureas can be used in the control of plant pathogens like *Penicillum expansum* and *Fusarium oxysporum* [18]. 1,3-Dialkyl or diaryl thioureas exhibited significant antifungal activity against *Pyricularia oryzae* and *Drechslera oryzae* [19]. Cao *et al*. reported that *γ*-aryl-1*H*-1,2,4-triazole derivatives showed potent antifungal activity against *Fusarium oxysporium*, *Rhizoctonia solani*, *Botrytis cinerea*, *Gibberella zeae*, *Dothiorella gregaria*, and *Colletotrichum gossypii* species [20,21]. Crank *et al.* [22] reported for the first time the antifungal activity of 2-aminooxazole thiourea derivatives against *Botrytis cinerea*. 

Data on the biological activity of new thiourea and urea derivatives bearing 1,2,4-triazole moieties is scanty. Considering the biological activities of these compounds, in this work a series of new thiourea and urea derivatives bearing 1,2,4-triazole moieties were prepared and evaluated for their antifungal activity against several filamentous fungal plant pathogens, mosquito (*Aedes aegypti*) larvicidal and biting deterrent activity and cytotoxicity and anti-inflammatory activity in some human cell lines.

## 2. Results and Discussion

### 2.1. Synthesis

**Scheme 1:**
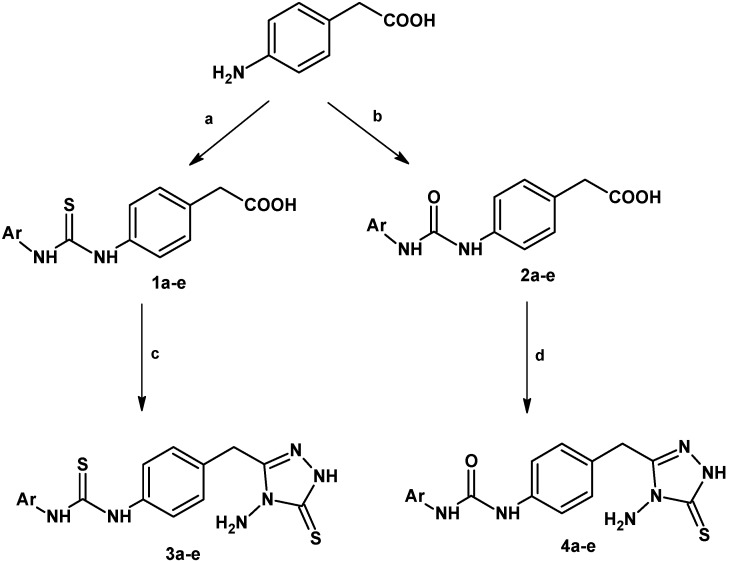
General synthetic route for title compounds **1a**–**e**, **2a**–**e**, **3a**–**e** and **4a**–**e**.

A series of new thiourea and urea derivatives bearing 1,2,4-triazole rings were prepared according to Scheme 1. The thiourea derivatives **1a**–**e** were previously synthesized and reported by Celen *et al.* [23]. Compounds **2a**–**e** were prepared by refluxing equimolar amounts of 4-(aminophenyl)acetic acid and various isocyanates in acetone. The reactions of compounds **1a**–**e** and **2a**–**e** with thio-carbohydrazide in an oil bath afforded the corresponding 1,2,4-triazoles **3a**–**e** and **4a**–**e**. All compounds were isolated in satisfactory yields (45–83%) and readily purified by recrystallization from acetonitrile. The purity of the compounds was checked by TLC and elemental analyses. The chemical structures of all compounds were characterized by various spectroscopic methods, and both the analytical and spectral data of all the synthesized compounds were in full agreement with the proposed structures. Physical and chemical properties of all compounds are presented in Table 1.

*Reagents and Conditions*: (**a**) Ar-NCS, 100 °C; (**b**) Ar-NCO, 100 °C; (**c**) NH_2_NHCSNHNH_2_, 130–140 °C; (**d**) NH_2_NHCSNHNH_2_, 130–140 °C.

**Table 1:** Structure and physical data of compounds **1a**–**e**, **2a**–**e**, **3a**–**e** and **4a**–**e**.

Compd.	Ar	M.P. (°C)	M.F.	M.W.	Yield (%)
**1a**	2,4,6-Cl-C_6_H_2_	214–215	C_15_H_11_Cl_3_N_2_O_2_S	389.68	50.5
**1b**	2,6-Cl_2_-C_6_H_4_	206–207	C_15_H_13_ClN_2_O_2_S	320.79	48.3
**1c**	4-CH_3_S-C_6_H_4_	193–194	C_16_H_16_N_2_O_3_S	316.37	61.1
**1d**	4-CF_3_-C_6_H_5_	240–241	C_15_H_13_N3O4S	331.34	45.5
**1e**	4-NO_2_-C_6_H_5_	200–201	C_15_H_13_FN_2_O_2_S	304.34	67.2
**2a**	2,4,6-Cl-C_6_H_2_	276–277	C_15_H_11_Cl_3_N_2_O_3_	373.62	77.3
**2b**	2,6-Cl_2_-C_6_H_4_	258–259	C1_5_H_13_ClN_2_O_3_	304.73	78.0
**2c**	4-CH_3_S-C_6_H_4_	234–235	C_16_H_16_N_2_O_4_	300.31	83.5
**2d**	4-CF_3_-C_6_H_5_	250–251	C_15_H_13_N_3_O_5_	315.28	67.4
**2e**	4-NO_2_-C_6_H_5_	245–246	C_15_H_13_FN_2_O_3_	288.27	80.9
**3a**	2,4,6-Cl-C_6_H_2_	227–228	C_16_H_13_Cl_3_N_6_S_2_	459.80	46.4
**3b**	2,6-Cl_2_-C_6_H_4_	206–207	C_16_H_15_ClN_6_S_2_	390.85	50.8
**3c**	4-CH_3_S-C_6_H_4_	228–230	C_17_H_18_N_6_OS_2_	386.56	59.3
**3d**	4-CF_3_-C_6_H_5_	240–241	C_15_H_17_N_7_O_2_S_2_	401.47	55.4
**3e**	4-NO_2_-C_6_H_5_	234–236	C_16_H_15_FN_6_S_2_	374.46	52.2
**4a**	2,4,6-Cl-C_6_H_2_	237–238	C_16_H_13_Cl_3_N_6_OS	443.74	58.0
**4b**	2,6-Cl_2_-C_6_H_4_	258–259	C_16_H_15_ClN_6_OS	374.85	55.9
**4c**	4-CH_3_S-C_6_H_4_	180–182	C_17_H_19_N_6_O_2_S	371.43	51.8
**4d**	4-CF_3_-C_6_H_5_	214–216	C_16_H_15_N_7_O_3_S	385.40	50.2
**4e**	4-NO_2_-C_6_H_5_	240–241	C_16_H_15_FN_6_OS	358.39	48.8

In the IR spectra of compounds **3a**–**e**, two C=S stretching bands due to the thiourea and thioxo groups were seen at 1320–1350 cm^−1^ and 1234–1294 cm^−1^. In the ^1^H-NMR spectrum, the carboxylic acid O-H peaks were observed as singlets at 11.98–12.58 ppm for compounds **1a**–**e** [23], but were absent in the spectra of the 1,2,4-triazole derivatives **3a**–**e** due to the ring closure. The N-H peaks of the triazole rings were seen at 11.70–12.30 ppm as singlets and the thiourea N-H peaks were observed at 7.83–9.79 ppm as two singlets. In the IR spectra of compounds **2a**–**e**, two C=O stretching bands due to the carboxylic acid and urea groups were detected at 1629–1697 cm^−1^. Urea N-H peaks were seen at 8.22–9.33 ppm as two singlets. The carboxylic acid O-H peaks were observed at 12.13–12.24 ppm as singlets, but these peaks disappeared in the spectra of 1,2,4-triazole derivatives **4a**–**e** due to the ring closure. The N-H peaks of the triazole rings were seen at 12.40-13.48 ppm as a singlet but some compounds (e.g., **4a**, **4b**) have no triazole ring-related N-H peaks due to deuterium exchange with the NMR solvent. The other aliphatic and aromatic protons were detected in the expected regions for all compounds. Mass spectra (MS-ES) of compounds showed [MH]^+^ peaks in agreement with their molecular formulae.

### 2.2. Biological Activity

**Figure 1:**
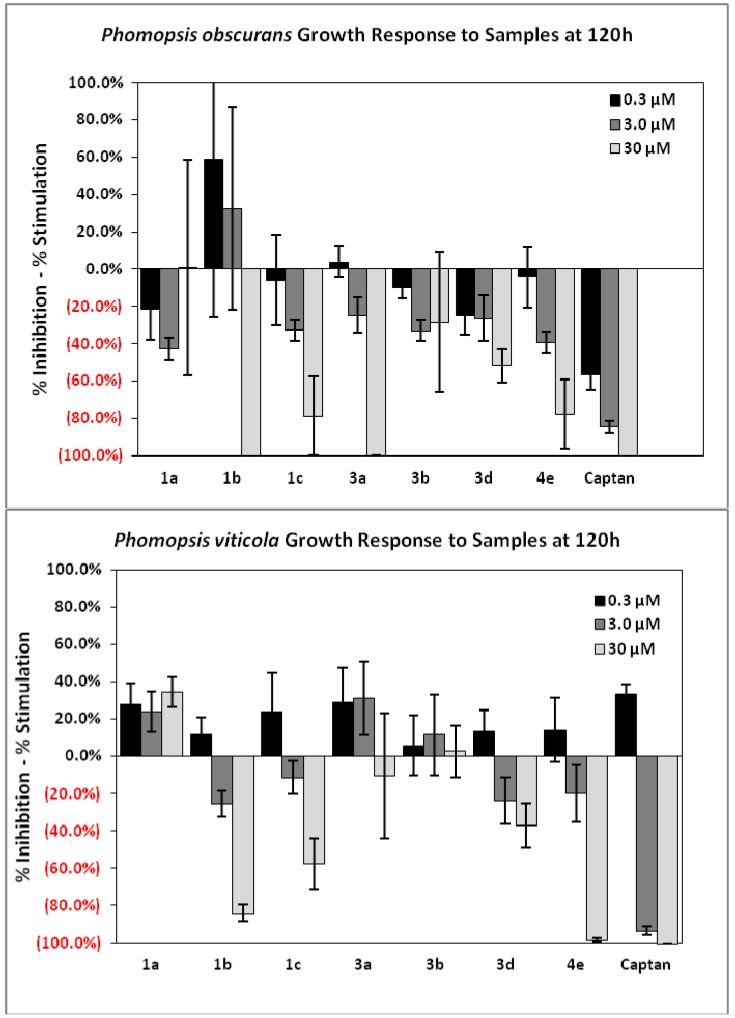
Growth inhibition of *Phomposis obscurans* and *P. viticola* after 120 h using a 96 well microdilution broth assay in a dose response with the commercial fungicide standard captan as reference.

**Figure 2:**
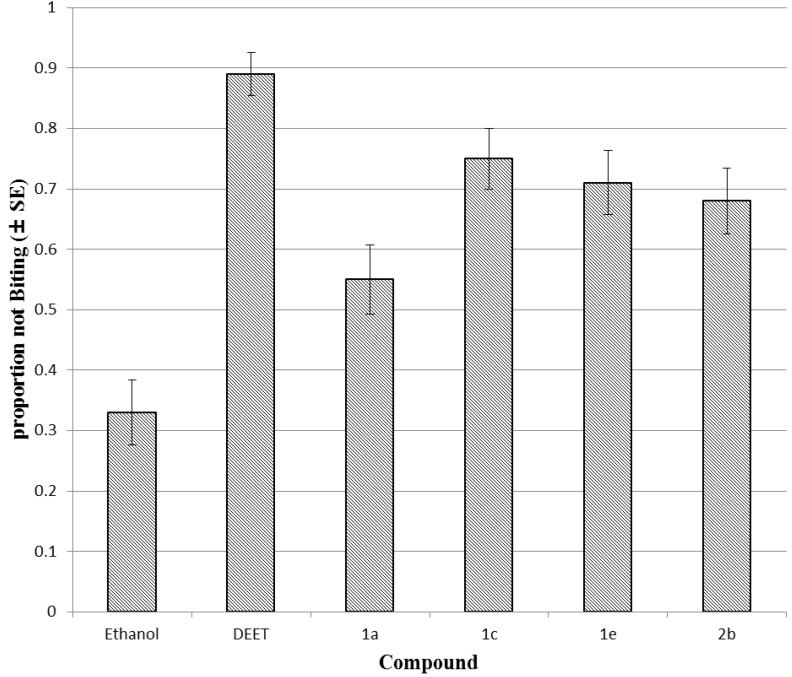
Biting deterrent effects of DEET **1a**, **1c** and **1e** and **2b** at 25 nmol/cm^2^ against *A. aegypti.*

All compounds were evaluated for growth inhibition of filamentous plant pathogenic fungi belonging to genera *Colletotrichum*, *Botrytis*, *Fusarium* and *Phomopsis* using 96-well micro-dilution broth assays. Microbioassay results indicated that the thiourea derivatives **1b**, **1c**, **3a** and **3d** bearing 2,6-dichloro-, 2,4,6-trichloro-, 4-methysulfanyl- and 4-trifluoromethyl- groups on the phenylthiourea ringand the urea derivative **4e** having a 4-nitro group were the most active against *Phomopsis obscurans* and *P. viticola.* Compounds **1b**, **3a**, and **1c** and **4e** at 30 μM inhibited the growth of *P.obscurans* by 100% and 80%, respectively, after 120 h exposure, whereas **1b**, **3d** and **4e** showed 80%, 60% and 80% growth inhibition, respectively, at 30 μM, respectively (Figure 1). Compound **4e** showed 100% growth inhibition of *P.viticola* at 30 μM, whereas **1b** and **1c** inhibited its growth by 80% and 60%, respectively (Figure 2). Compounds **1b**, **3a** and **4e** having 2,6-dichloro-, 2,4,6-trichloro- and 4-nitro-groups on the phenylthiourea ring showed fungal growth inhibition that was similar to that of 30 μM of the positive control captan, a well known multisite inhibitor fungicide with no systemic activity, used as a commercial protectant fungicide to prevent anthracnose diseases in fruits and ornamentals [24]. Tested compounds showed a very weak activity against *Colletotrichum*, *Botrytis* and *Fusarium* species.

In biting deterrent bioassays with *A*. *aegypti*, compounds **1a**, **2b**, **1e** and **1c** showed significantly higher biting deterrent activity (Figure 2) than the control, while the activity in the other compounds was similar to that of the ethanol control. Compound **1c** bearing a 2,4,6-trichloro group had the highest activity, with a portion not biting (PNB) value of 0.75, followed by **1e**, **2b** and **1a** having 4-nitro-, 2,6-dichloro- and 2,4,6-trichloro-groups, with PNB values of 0.71, 0.68 and 0.55, respectively, at 25 nmol/cm^2^. However, biting deterrent activity in all these compounds was significantly lower than that of the reference compound DEET (PNB = 0.89).

In order to discover new mosquito toxicants, synthesized compounds were also screened against 1 d old larvae. The three compounds **1c**, **3d** and **3e** which showed larvicidal activity in preliminary screening were selected for further study in dose response bioassays. Dose mortality data of these thiourea compounds are presented in Table 2. 

**Table 2:** Toxicity of substituted urea and thiourea 1,2,4-triazole derivatives **1c**, **3d** and **3e** against 1-d old larvae of *Aedes aegypti* at 24-h post treatment*.*

Compounds	LD_50_ (95% CI) *	LD_90_ (95% CI) *	Chi square	DF
**1c**	118.8 (105.3–135.2)	216.4 (182.4–280.4)	61.7	38
**3d**	67.4 (59.0–77.0)	139.4 (116.4–180.6)	75.0	38
**3e**	165.6 (141.7–205.2)	370.95 (278.5–619.3)	40.3	38

* LD values are in ppm; 95% CI = 95% confidential intervals.

Compound **3d** carrying a 4-trifluorophenyl group (LD_50_ value of 67.9 ppm) showed the highest mortality, followed by **1c** having a 4-methylsulfanylphenyl group on the thiourea moiety (LD_50_ = 118.8 ppm) and **3e** with a 4-nitrophenyl moiety (LD_50_ = 165.6). The comparison of larvicidal activity of thiourea and urea derivatives showed that thiourea derivatives were much more potent than the corresponding urea derivatives. There was not not much increase in mortality at 48 h post treatment.

All synthesized compounds were tested for their cytotoxicity and anti-inflammatory activity in cellular assays. No anti-inflammatory activity was observed against NF-κB mediated transcription induced by phorbol myristate acetate (PMA) in human chondrosarcoma cells up to the highest tested concentration of 25 μg/mL and no cytotoxicity was observed against mammalian kidney cells (Vero and LLC-PK_11_) or human solid tumor cells (SK-MEL, SK-OV-3, BT-549 and KB) up to a highest concentration of 25 μg/mL, except for compound 3e having 4-nitro group, which showed mild cytotoxicity with IC_50_ values in the range of 11.0–25.0 μg/mL towards most of the tested cell lines. 

## 3. Experimental

### 3.1. General

Reactions were monitored by thin layer chromatography (TLC) performed on 60 F-254 silica gel plates with visualization by UV-light using chloroform and methanol as solvent system and purity of the products was checked by High Performance Liquid Chromatography (HPLC, Agilent Technologies, Palo Alto, CA, USA). Melting points were determined on a SMP II apparatus (Schorpp Geaetetechnik Gehrden, Germany). The UV spectra were measured with a Shimadzu UV–2100 S (Shimadzu, Kyoto, Japan).The IR spectra were recorded on a Shimadzu FTIR 8400S spectrometer (Shimadzu, Kyoto, Japan). ^1^H-NMR spectra were recorded at 400 MHz on Bruker Avance-DPX-400 spectrometer (Bruker Spectrospin, Billerica, MA, USA) in DMSO-d_6_. Chemical shifts were recorded in parts per million (ppm) downfield from TMS. The splitting patterns of ^1^H-NMR were designated as follows: s: singlet, d: doublet, t: triplet, q: quartet, m: multiplet. Mass spectra were recorded on an Agilent 1100 LC-MS series system (Agilent Technologies, Palo Alto, CA, USA) in the electrospray mode. Elemental analysis was performed on a Leco CHNS-932 analyzer (Leco, Michigan, MI, USA). All chemicals and solvents were procured from Merck or Aldrich (Darmstad and Steinheim, Germany). The thiourea derivatives **1a**–**e** were prepared by reacting 4-(aminophenyl)acetic acid with the corresponding isothiocyanate. Spectroscopic characterisation of **1a**–**e** has been previously reported [23]. 

#### 3.1.1. General Procedure for the Preparation of 2a–e

4-(Aminophenyl)acetic acid (0.500 g, 3.3 mmol) was dissolved in acetone (5 mL) at 100 °C. Then, a solution of the corresponding isocyanate (3.3 mmol) in acetone (5 mL) was added in three portions separated by 30 min. After 6–8 h, the reaction was finalized according to TLC monitoring. Solid material was filtered off and recrystallized from a suitable solvent.

*(4-{[(2,4,6-Trichlorophenyl)carbamoyl]amino}phenyl)acetic acid* (**2a**). UV λ_max._ (EtOH) nm (log ε): 251 (4.45). IR (ν_max,_ cm^−1^): 3286 (N-H, O-H), 3286 (C-H), 1687, 1651 (C=O), 1543, 1510 (N-H). ^1^H-NMR δ: 3.48 (2H, s, -CH_2_), 6.99–7.83 (8H, m, Ar*-*H), 8.22 (1H, s, -NH-), 8.94 (1H, s, -NH-), 12.24 (1H, s, OH). Anal. Calcd. for C_15_H_11_Cl_3_N_2_O_3_; C: % 48.22; H: % 2.97; N: % 7.50 Found: C: % 47.95; H: % 3.00; N: % 7.30. MS-ES (*m/z*): 374.62 (MH^+^). 

*(4-{[(2,6-Dichlorophenyl)carbamoyl]amino}phenyl)acetic acid* (**2b**). UV λ_max._ (EtOH) nm (log ε): 251 (4.41). IR (υ_max,_ cm^−1^): 3252 (N-H, O-H), 3016 (C-H), 1697, 1651 (C=O), 1573, 1545 (N-H). ^1^H-NMR δ: 3.49 (2H, s, -CH_2_), 7.09–7.60 (7H, m, Ar*-*H), 8.18 (1H, s, -NH-), 8.91 (1H, s, -NH-), 12.24 (1H, s, OH). Anal. Calcd. for C_15_H_12_Cl_2_N_2_O_3_; C: % 53.12; H: % 3.57; N: % 8.26 Found: C: % 52.84; H: % 3.55; N: % 8.15. MS-ES (*m/z*): 340.18 (MH^+^). 

*4-{[(4-(Methylsulfanyl)phenyl)carbamoyl]amino}phenyl)acetic acid* (**2c**). UV λ_max._ (EtOH) nm (log ε): 279 (3.12). IR (ν_max,_ cm^−1^): 3290 (N-H, O-H), 3007 (C-H), 1695,1629 (C=O), 1550 (N-H). ^1^H-NMR δ: 3.35 (3H, s, CH_3_), 3.54 (2H, s, -CH_2_), 7.02–7.62 (8H, m, Ar-H), 8.63 (1H, s, -NH-), 8.70 (1H, s, -NH-), 12.24 (1H, s, OH). Anal. Calcd. for C_16_H_16_N_2_O_3_S; C: % 60.74; H: % 5.10; N: % 8.85, S: 10.14 Found: C: % 60.54; H: % 5.09; N: % 8.73, S: 10.00. MS-ES (*m/z*): 317.37 (MH^+^).

*(4-{[(4-Trifluoromethylphenyl)carbamoyl]amino}phenyl)acetic acid* (**2d**). UV λmax. (EtOH) nm (log ε): 261 (4.06). IR (νmax, cm−1): 3304 (N-H, O-H), 3304 (C-H), 1697,1630 (C=O), 1552, 1512 (N-H). 1H-NMR δ: 3.48 (2H, s, -CH2), 6.81–7.42 (8H, m, Ar-H), 8.43 (1H, s, -NH-), 8.53 (1H, s, -NH-), 12.21 (1H, s, OH). Anal. Calcd. for C16H13F3N2O3; C: % 56.81; H: % 3.87; N: % 8.28 Found: C: % 56.60; H: % 3.72; N: % 8.16. MS-ES (m/z): 339.28 (MH+).

*(4-{[(4-Nitrophenyl)carbamoyl]amino}phenyl)acetic acid* (**2e**). Compound **2e** was previously synthesized by Deny *et al.* [25]. UV λ_max._ (EtOH) nm (log ε): 249 (4.08). IR (ν_max,_ cm^−1^): 3367 (N-H, O-H), 3308 (C-H), 1672, 1640 (C=O), 1556,1496 (N-H). ^1^H-NMR δ: 3.25 (2H, s, -CH_2_), 7.11–8.30 (8H, m, Ar-H), 8.70 (1H, s, -NH-), 9.33 (1H, s, -NH-), 12.13 (1H, s, OH). Anal. Calcd. for C_15_H_13_N_3_O_5_: C: % 57.14; H: % 4.16; N: % 13.33 Found: C: % 57.27; H: % 4.10; N: % 13.38. MS-ES (*m/z*): 316.28 (MH^+^).

#### 3.1.2. General Procedure for the Preparation of **3a**–**e** and **4a**–**e**

Equimolar mixtures of thiocarbohydrazide (0.1 mol) and thiourea or urea derivatives **1a**–**e**, **2a**–**e** were heated in an oil-bath at 130–140 °C for 2–3 h. The fused mass obtained was dispersed with hot water to obtain the new thiourea and urea derivatives **3a**–**e**, **4a**–**e** containing 1,2,4-triazole moieties. The products were recrystallized from methanol.

*1-{4-[(4-Amino-5-thioxo-4,5-dihydro-1H-1,2,4-triazole-3-yl)methyl]phenyl}-3-(2,4,6-trichlorophenyl)-thiourea* (**3a**). UV λ_max._ (EtOH) nm (log ε): 258 (3.97). IR (ν_max,_ cm^−1^): 3252 (N-H), 3020 (C-H) 1651 (C=N), 1600, 1510 (N-H), 1345, 1246 (C=S), 1182 (C-N). ^1^H-NMR δ: 3.20 (2H, s, -CH_2_), 5.48 (2H, s, NH_2_), 7.20–7.46 (6H, m, Ar*-*H), 7.83 (1H, s, -NH-), 8.55 (1H, s, -NH-), 11.70 (1H, s, triazole NH). Anal. Calcd. for C_16_H_13_Cl_3_N_6_S_2_; C: % 41.79; H: % 2.85; N: % 18.28; S: % 13.95 Found: C: % 41.64; H: % 3.09; N: % 18.25; S: % 13.72. MS-ES (*m/z*): 460.80 (MH^+^).

*1-{4-[(4-Amino-5-thioxo-4,5-dihydro-1H-1,2,4-triazole-3-yl)methyl]phenyl}-3-(2,6-dichlorophenyl)-thiourea* (**3b**). UV λ_max._ (EtOH) nm (log ε): 278 (3.51). IR (ν_max_, cm^−1^): 3194 (N-H), 3012 (C-H), 1650 (C=N), 1541 (N-H), 1350, 1240 (C=S), 1180 (C-N). ^1^H-NMR δ: 3.47 (2H, s, -CH_2_), 5.50 (2H, s, NH_2_), 7.15–7.59 (7H, m, Ar*-*H), 9.68 (1H, s, -NH-), 9.79 (1H, s, -NH-), 11.80 (1H, s, triazole NH). Anal. Calcd. for C_16_H_14_Cl_2_N_6_S_2_; C: % 45.18; H: % 3.32; N: % 19.76; S: % 15.08. Found: C: % 45.10; H: % 3.30; N: % 19.66; S: % 15.00. MS-ES (*m/z*): 426.36 (MH^+^).

*1-{4-[(4-Amino-5-thioxo-4,5-dihydro-1H-1,2,4-triazole-3-yl)methyl]phenyl}-3-(4-(methylsulfanyl)-phenyl)thiourea* (**3c**). UV λ_max._ (EtOH) nm (log ε): 282 (3,30). IR (ν_max_, cm^−1^): 3220 (N-H), 3026 (C-H), 1537 (N-H), 1345, 1234 (C=S), 1178 (C-N). ^1^H-NMR δ: 3.30 (3H, s, CH_3_), 3.49 (2H, s, -CH_2_), 5.10 (2H, s, NH_2_), 6.90–7.40 (8H, m, Ar*-*H), 9.40 (1H, s, -NH-), 9.46 (1H, s, -NH-), 12.00 (1H, s, triazole NH). Anal. Calcd. for C_17_H_18_N_6_OS_2_; C: % 50.72; H: % 4.51; N: % 20.88; S: % 23.90. Found: C: % 50.27; H: % 4.70; N: % 20.69; S: % 23.92. MS-ES (*m/z*): 403.56 (MH^+^).

*1-{4-[(4-amino-5-thioxo-4,5-dihydro-1H-1,2,4-triazole-3-yl)methyl]phenyl}-3-(4-(trifluoromethyl)-phenyl)thiourea* (**3d**). UV λ_max._ (EtOH) nm (log ε): 257 (4.62). IR (ν_max_, cm^−1^): 3288 (N-H, C-H), 1697 (C=N), 1627, 1552 (N-H), 1346, 1294 (C=S), 1188 (C-N). ^1^H-NMR δ: 3.41 (4H, d, -NH_2_, -CH_2_), 7.05–7.52 (8H, m, Ar-H), 8.45 (1H, s, -NH-), 8.69 (1H, s, -NH-), 12.30 (1H, s, triazole NH). Anal. Calcd. for C_17_H_15_F_3_ N_6_S_2_; C: % 48.10; H: % 3.56; N: % 19.80; S: % 15.11 Found: C: % 50.29; H: % 4.12; N: % 22.50; S: % 17.17. MS-ES (*m/z*): 425.47 (MH^+^).

*1-{4-[(4-Amino-5-thioxo-4,5-dihydro-1H-1,2,4-triazole-3-yl)methyl]phenyl}-3-(4-nitrophenyl)thiourea* (**3e**). UV λ_max._ (EtOH) nm (log ε): 246 (4.06). IR (ν_max_, cm^−1^): 3280 (N-H), 3023 (C-H), 1593 (C-NO_2_ assymetrical stretching), 1494 (N-H), 1320, 1298 (C=S), 1170 (C-N). ^1^H-NMR δ:3.55 (2H, s, -CH_2_), 5.25 (2H, s, NH_2_), 7.11–8.36 (8H, m, Ar-H), 9.20 (1H, s, -NH-), 9.40 (1H, s, -NH-), 12.20 (1H, s, triazole NH). Anal. Calcd. for C_16_H_15_N_7_O_2_S_2_; C: % 47.87; H: % 3.77; N: % 24.42; S: % 15.97. Found: C: % 47.80; H: % 3.74; N: % 24.40; S: % 15.96. MS-ES (*m/z*): 402.47 (MH^+^).

*1-{4-[(4-Amino-5-thioxo-4,5-dihydro-1H-1,2,4-triazole-3-yl)methyl]phenyl}-3-(2,4,6-trichlorophenyl)-urea* (**4a**). UV λ_max._ (EtOH) nm (log ε): 253 (3.24). IR (ν_max,_ cm^−1^): 3271 (N-H, C-H), 1651 (C=O), 1595 (C=N), 1539, 1508 (N-H), 1284–1253 (C=S), 1182 (C-N). ^1^H-NMR δ: 4.96 (4H, s, -NH_2_, -CH_2_), 7.15–7.79 (6H, m, Ar-H), 9.12 (1H, s, -NH-), 9.63 (1H, s, -NH-). Anal. Calcd. for C_16_H_13_Cl_3_N_6_OS; C: % 43.31; H: % 2.95; N: % 18.94; S: % 7.23 Found: C: % 42.95; H: % 2.91; N: % 19.03; S: % 7.24. MS-ES (*m/z*): 444.74 (MH^+^).

*1-{4-[(4-Amino-5-thioxo-4,5-dihydro-1H-1,2,4-triazole-3-yl)methyl]phenyl}-3-(2,6-dichlorophenyl)-urea* (**4b**). UV λ_max._ (EtOH) nm (log ε): 263 (4.60). IR (ν_max,_ cm^−1^): 3300 (N-H, C-H), 1699 (C=O), 1633 (C=N), 1591, 1541 (N-H), 1228 (C=S), 1089 (C-N). ^1^H-NMR δ: 3.47 (4H, s, -NH_2_, -CH_2_), 7.10–7.58 (7H, m, Ar*-*H), 9.29 (1H, s, -NH-), 9.48 (1H, s, -NH-). Anal. Calcd. for C_16_H_14_Cl_2_N_6_OS_2_; C: % 45.18; H: % 3.45; N: % 20.53; S: % 7.83 Found: C: % 47.00; H: % 3.42; N: % 20.50; S: % 7.92. MS-ES (*m/z*): 410.29 (MH^+^).

*1-{4-[(4-Amino-5-thioxo-4,5-dihydro-1H-1,2,4-triazole-3-yl)methyl]phenyl}-3-(4-methylsulfanyl-phenyl)urea* (**4c**). UV λ_max._ (EtOH) nm (log ε): 261 (4.06). IR (ν_max,_ cm^−1^): 3304 (N-H, O-H), 3304 (C-H), 1697, 1633 (C=O), 1552,1512 (N-H). ^1^H-NMR δ: 3.32 (3H, s, CH_3_), 3.48 (2H, s, -CH_2_), 5.20 (2H, s, -NH_2_), 6.81–7.42 (8H, m, Ar*-*H), 8.43 (1H, s, -NH-), 8.53 (1H, s, -NH-), 12.40 (1H, s, triazole NH). Anal. Calcd. for C_17_H_18_N_6_OS; C: % 52.83; H: % 4.69; N: % 21.70; S: 16.59. Found: C: % 52.70; H: % 4.49; N: % 21.67; S: 16.50. MS-ES (*m/z*): 387.49 (MH^+^).

*1-{4-[(4-Amino-5-thioxo-4,5-dihydro-1H-1,2,4-triazole-3-yl)methyl]phenyl}-3-(4-trifluoromethyl-phenyl)urea* (**4d**). UV λ_max._ (EtOH) nm (log ε): 258 (4.33). IR (ν_max,_ cm^−1^): 3290 (N-H), 1699 (C=O), 1556 (C=N), 1510, 1494 (N-H), 1211-1186 (C=S), 1151, 1095 (C-N). ^1^H-NMR δ: 3.42 (4H, d, -NH_2_, -CH_2_), 7.02–7.66 (8H, m, Ar*-*H), 8.65 (2H, d, -NH-, -NH-), 12.50 (1H, s, triazole NH). Anal. Calcd. for C_17_H_15_F_3_ N_6_OS; C: % 50.00; H: % 3.70; N: % 20.58; S: % 7.85 Found: C: % 50.10; H: % 3.67; N: % 20.50; S: % 7.89. MS-ES (*m/z*): 409.40 (MH^+^).

*1-{4-[(4-Amino-5-thioxo-4,5-dihydro-1H-1,2,4-triazole-3-yl)methyl]phenyl}-3-(4-nitrophenyl)urea* (**4e**). UV λ_max._ (EtOH) nm (log ε): 253 (3,36). IR (ν_max,_ cm^−1^): 3290 (N-H, C-H), 1695 (C=O), 1629 (C=N), 1589, 1545 (N-H), 1296 (C=S), 1111 (C-N). ^1^H-NMR δ: 3.48 (2H, s, -CH_2_), 5.40 (2H, s, -NH_2_), 7.01–7.62 (8H, m, Ar*-*H), 8.52 (1H, s, -NH-), 9.93 (1H, s, -NH-), 13.48 (1H, s, triazole NH). Anal. Calcd. for C_16_H_15_FN_7_O_3_S; C: % 49.86; H: % 3.92; N: % 25.44; S: % 8.32 Found: C: % 50.08; H: % 4.08; N: % 25.37; S: % 8.26. MS-ES (*m/z*): 386.40 (MH^+^).

### 3.2. Biological Activity

#### 3.2.1. Antifungal Activity

A standardized 96-well micro-dilution broth assay as developed by Wedge and Kuhajek [26] was used to evaluate the antifungal activity of the compounds towards *Botrytis cinerea*, *Colletotrichum acutatum*, *C. fragariae*, *C. gloeosporioides*, *P.* *viticola*, *P. obscurans* and *Fusarium oxysporum*. The fungicide captan was used as an internal fungicide standard in all assays. Each fungus was challenged in duplicate in a dose-response format using test compounds where the final treatment concentrations were 0.3, 3.0 and 30.0 μM. Microtiter plates (Nunc MicroWell, untreated, Roskilde, Denmark) were covered with a plastic lid and incubated in a growth chamber as described previously [27]. Fungal growth was then evaluated by measuring absorbance of each well at 620 nm using a microplate photometer (Packard Spectra Count, Packard Instrument Co., Downers Grove, IL, USA). Sixteen wells containing broth and inoculum served as positive growth controls, eight wells containing solvent at the appropriate concentration and broth without inoculum were used as negative growth controls. The experiments were repeated at three times over a period of time. Mean absorbance values and standard errors were used to evaluate fungal growth at 48 h and 72 h except for *P. obscurans* and *P.* *viticola* where the data were recorded at 144 h. Means for percent inhibition of each fungus at each dose of test compound relative to the untreated positive growth controls were used to evaluate fungal growth. The SAS Proc ANOVA module [28] was used to identify significant factors, and Fisher’s protected LSD was used to separate means [29]. 

#### 3.2.2. Mosquito Larval Bioassay

*Aedes aegypti* (L.) used in these studies were from a laboratory colony maintained since 1952 at the Mosquito and Fly Research Unit at Center for Medical, Agricultural and Veterinary Entomology, USDA-ARS, Gainesville, FL, USA. This colony is maintained using standard procedures [30]. Bioassays to determine the larvicidal activity of urea and thiourea derivatives against *A. aegypti* were conducted by using the system described by Pridgeon *et al.* [31]. In brief, the eggs were hatched under vacuum (about 1 h) by placing a piece of a paper towel with eggs in a cup filled with 100 mL of deionized water containing small quantity of larval diet. Larvae were removed from vacuum and held overnight in the cup in a temperature-controlled room maintained at a temperature of 27 ± 2 °C and 60 ± 10% RH at a photoperiod regimen of 12:12 (L:D) h. Five 1-d-old first instar *A. aegypti* were added to each well of 24-well plates in a droplet of water using a disposable 22.5-cm Pasteur pipette by placing on an illuminated light box. One mL deionized water was added to each well using a Finnpipette stepper (Thermo Fisher, Vantaa, Finland). Fifty µL of larval diet (2% slurry of 3:2 beef liver powder (Now Foods, Bloomingdale, IL, USA) and brewer’s yeast (Lewis Laboratories Ltd., Westport, CT, USA) were then added to each well. All chemicals to be tested were diluted in dimethyl sulfoxide (DMSO). Eleven microliters of the test chemical was added to the labeled wells, and in control treatments 11 µL of DMSO alone was added. After the treatment, the plates were swirled in clockwise and counter clockwise motions and front and back and side to side five times to ensure even mixing of the chemicals. Larval mortality was recorded 24- and 48-h after treatment. Larvae that showed no movement in the well after manual disturbance of water by a pipette tip were recorded as dead. A series of concentrations were used in each treatment to get a range of mortality. Treatments were replicated 15 times for each compound. LD_50_ values for larvicidal data were calculated by using the SAS Proc Probit module [28]. Control mortality was corrected by using Abbott’s formula.

#### 3.2.3. Mosquito Biting Bioassays

Experiments were conducted using a six-celled *in vitro* Klun & Debboun (K & D) module bioassay system as developed by Klun *et al.* [32] for quantitative evaluation of bite deterrent properties of candidate compounds. The term deterrent refers to a chemical that inhibits feeding when present in a place where the insects feed in its absence and the repellent is a chemical that causes insects to make oriented movement away from its source (Dethier [33]). Briefly, the assay system consists of a six well reservoir with each of the 3 cm × 4 cm wells containing 6 mL of blood. As described by Ali *et al.* [34], a feeding solution consisting of CPDA-1 and ATP was used instead of blood. *N*,*N*-diethyl-*meta*-toluamide (DEET) was obtained from Sigma Aldrich (St. Louis, MO, USA) and used as a positive control. Molecular biology grade ethanol was obtained from Fisher Scientific Chemical Co. (Fairlawn, NJ, USA). All synthesized compounds (Table 1) were screened in this study and DEET at 25 nmol/cm^2^ was used as positive control. All the treatments were freshly prepared in ethanol at the time of bioassay.

The temperature of the solution in the reservoirs was maintained at 37.5 °C by continuously passing the warm water through the reservoir using a circulatory bath. The reservoirs were covered with a layer of collagen membrane. The test compounds were randomly applied to six 4 cm × 5 cm areas of organdy cloth and positioned over the membrane-covered CPDA-1+ATP solution with a separator placed between the treated cloth and the six-celled module. A six celled K & D module containing five females per cell was positioned over cloth treatments covering the six CPDA-1+ATP solution membrane wells, and trap doors were opened to expose the treatments to these females. The number of mosquitoes biting through cloth treatments in each cell was recorded after a 3 min exposure and mosquitoes were prodded back into the cells. These mosquitoes were then squashed to determine the number which has actually engorged the solution. A replicate consisted of six treatments: four test compounds, DEET (a standard biting deterrent) and ethanol treated cloth as solvent control. A set of five replications was conducted on different days using a new batch of females in each replication. Treatments were replicated 15 times.

#### 3.2.4. Cytotoxicity Activity

*In vitro* cytotoxicity was determined against a panel of human cancer cell lines (SK-MEL, KB, BT-549, SK-OV-3) as well as noncancerous pig kidney epithelial (LLC-PK_11_) and monkey kidney fibroblast (VERO) cells. The assay was performed in 96-well tissue culture-treated microplates. Cells were seeded to the wells of the plate (25,000 cells/well) and incubated for 24 hours. Samples were added and again incubated for 48 h. The number of viable cells was determined using the supravital dye Neutral Red according to the procedure described earlier [35]. Doxorubicin was used as control drug for cytotoxicity.

#### 3.2.5. Anti-Inflammatory Activity

Inhibition of NF-*κ*B mediated transcription was determined in human chondrosarcoma (SW1353) cells by a reporter gene assay as described earlier [36,37]. In brief, at about 75% confluency, cells were harvested and transfected with NF-*κ*B reporter luciferase plasmid construct at 160 V and one 70-ms pulse in a BTX Electro Square Porator T 820. Transfected cells were plated in 96-well plates (1 × 10^5^ cells/well) and incubated for 24 h. After 24 h, cells were treated with test samples and after 30 min treated with PMA (70 ng/mL) for the activation of NF-*κ*B and incubated for 8 h. After removing medium, cells were lysed by adding 40 µL of a 1:1 mixture of LucLite reagent and PBS containing 1 mM calcium and magnesium. Luciferase activity was measured as light output on a SpectraMax plate reader. Sp-1 was used as a control transcription factor to evaluate the toxicity of tested compounds in the same assay and parthenolide was used as a positive control as described earlier [37].

## 4. Conclusions

The molecules described in this study demonstrated no cytotoxicity and no anti-inflammatory activity, but some of them showed good antifungal activity against *Phomopsis* species. Based on the biological activity evaluation these compounds may not be useful as therapeutic or pharmaceutical agents. However, selective compounds exhibiting antifungal activity could have a potential to be developed as agrochemicals, and further work should be performed to modify their structure with an aim to increase their activity. *Phomopsis* appeared to be the most sensitive fungal species to the tested compounds. *Phomopsis* cane and leaf spot (*P. viticola*) causes serious economic losses to the vine grape production in the United States of America and Europe, while *P. obscurans* causes *Phomopsis* leaf blight and fruit rot of strawberry. Compound **3d** was the most active derivative among the tested compounds in a larvicidal assay against *A. aegypti.* The comparison of antifungal and insecticidal activities of thiourea and urea derivatives showed that thiourea derivatives bearing 2,6-dichlorophenyl, 2,4,6-trichlorophenyl, 4-nitophenyl, 4-trifluoromethylphenyl substituents were the active compounds. Thiourea compounds, **1b**, **1c**, **3a**, **3d**, **3e**, and urea compound **4e** show sufficient potential to warrant further modification to discover new antifungal and insecticidal molecules.
